# *In situ* single cell detection via microfluidic magnetic bead assay

**DOI:** 10.1371/journal.pone.0172697

**Published:** 2017-02-21

**Authors:** Fan Liu, Pawan KC, Ge Zhang, Jiang Zhe

**Affiliations:** 1 Department of Mechanical Engineering, University of Akron, Akron, Ohio, United States of America; 2 Department of Biomedical Engineering, University of Akron, Akron, Ohio, United States of America; The Ohio State University, UNITED STATES

## Abstract

We present a single cell detection device based on magnetic bead assay and micro Coulter counters. This device consists of two successive micro Coulter counters, coupled with a high gradient magnetic field generated by an external magnet. The device can identify single cells in terms of the transit time difference of the cell through the two micro Coulter counters. Target cells are conjugated with magnetic beads via specific antibody and antigen binding. A target cell traveling through the two Coulter counters interacts with the magnetic field, and have a longer transit time at the 1st counter than that at the 2nd counter. In comparison, a non-target cell has no interaction with the magnetic field, and hence has nearly the same transit times through the two counters. Each cell passing through the two counters generates two consecutive voltage pulses one after the other; the pulse widths and magnitudes indicating the cell’s transit times through the counters and the cell’s size respectively. Thus, by measuring the pulse widths (transit times) of each cell through the two counters, each single target cell can be differentiated from non-target cells even if they have similar sizes. We experimentally proved that the target human umbilical vein endothelial cells (HUVECs) and non-target rat adipose-derived stem cells (rASCs) have significant different transit time distribution, from which we can determine the recognition regions for both cell groups quantitatively. We further demonstrated that within a mixed cell population of rASCs and HUVECs, HUVECs can be detected *in situ* and the measured HUVECs ratios agree well with the pre-set ratios. With the simple device structure and easy sample preparation, this method is expected to enable single cell detection in a continuous flow and can be applied to facilitate general cell detection applications such as stem cell identification and enumeration.

## Introduction

Highly efficient cell identification is a vital task to advance current stem cell research,[1–5] cancer therapeutics,[6–9] and drug discovery.[10] Identification and isolation of pure stem cell population from various tissues at a high speed and a low cost can enable mass production of therapeutic cells for the next generation of cell therapy.[11,12] Accurate detection of rare cancer cells can dramatically improve early cancer screening and diagnosis. [7,13] Additionally, sensitive measurement of single cell responses to specific pharmaceuticals will greatly accelerate new drug discovery.[14,15] Traditional bulk cell detection methods including ELISA (enzyme-linked immunosorbent assay),[16,17] high throughput microscopy,[18] and magnetic resonance imaging[19] can detect cells by measuring the average optical or magnetic responses from a large cell population. However, these methods have limited sensitivity and resolution because of bulk measurements and cannot fulfill the growing need for highly efficient and sensitive cell detection.[20–24]

Identification and enumeration at single cell level significantly increase the sensitivity and specificity of cell detection. Fluorescence-activated cell sorting (FACS) is the most widely used technique to characterize single cell properties and count the specific cell numbers. FACS works by measuring the fluorescent signals from single cells, on a cell-by-cell basis.[10,25] Each cell is labeled with fluorescence tags corresponding to its surface antigens. When the cell is driven through the sensing zone and excited by a focused laser light, it emits out fluorescence light; the light wavelength and intensity indicate the specific cell antigen receptor type and density. This method can detect multiple fluorescence tags with a high throughput. However, to increase the optical signal strength and suppress the background noise, complex optical components (excitation light source/ filters/ detectors) and a delicate cell focusing system must be used. Therefore, the system is bulky, costly, and often difficult to access. In addition, this method typically requires a large number of cells (~10^5^ cells per run) and reagents, and is vulnerable to contaminations when processing infectious samples.[26]

To date, the impedance flow cytometry methods [27,28] have evolved from the basic Coulter counter[29,30] that measures cell size and counts to more advanced devices [28,31–34] that can differentiate specific cell types. However, these methods are still limited by their insufficient sensitivity in detecting the subtle differences of cell antigen expressions between the subpopulations of cells. [27,32]. Recently, microfluidic technologies and immunobinding approaches have been utilized for cell detection methods. Sohn et al. developed a microfluidic device to detect and count murine erythroleukemia (MEL) cells based on the cells’ transit time change, which is induced by cells’ interaction with the CD34 antibody functionalized microchannel.[35] However, to generate transit time change, this approach requires antibody modification of microchannel surface prior to cell analysis, which is difficult to perform within the microscale channels and hence limits the practical application. Moreover, the functionalized channel also has non-specific interaction with the non-target cells, resulting in an overlapped transit time distribution for the mixed cell population. Thus, by measuring the average transit time of a cell population, the device is unable to identify each single cell *in situ*.

Immuno-magnetic separation has been routinely used to isolate various cell populations. The target cells were tagged with magnetic particles via specific binding between antigen and antibody, and can be selectively extracted by an external magnetic field. In the immuno-magnetic separation, magnetic particle size and the external magnetic field gradient govern the target cell magnetic mobility and affect the separation efficiency.[36,37] Magnetic activated cell separation (MACS^®^) system typically uses small magnetic particles (~50 nm) and a high gradient magnetic separation (HGMS) column for fast and stable binding.[38,39] If larger magnetic beads (i.e. Dynabeads) are used, column free system allows direct cell separation in tubes [40–42] and microchannels.[43] In contrast to these methods relying on capturing target cells, magnetophoretic-based devices have also been developed to separate the target cells from the continuous flow.[44–51] By controlling the cells’ trajectory with a magnetic field,[49,52–56] target cells can be manipulated toward different fluid layers [50,55] or different exit channels.[48,49] However, the above-mentioned systems focus on high efficiency of cell sorting and separation; they are unable to identify, count and measure the size of cells from the heterogeneous population. To solve the problem, in our prior work, microfluidic devices consisting of two individual Coulter counters separated by a fluidic chamber were reported. An external magnetic field was applied to the fluidic chamber by a layer of magnetic beads on the chamber’s substrate or by an external permanent magnet. Only target cells were trapped or slowed down by the fluidic chamber because of the magnetic interactions. [57,58] By counting the pulse number difference of the two Coulter counters or measuring the average transit time change of a cell population, target cells ratio in a heterogeneous cell population can be estimated. However, because the volume of the fluidic chamber is three orders higher than that of the micro Coulter counter, the cells would flow along different paths/streamlines as they travel through the chamber. Hence their original flow sequence would be disrupted when they enter the 2nd counter. As a result, it is difficult to pair the voltage pulses generated by a single cell with the device in reference [57]; hence it cannot detect transit time change of a single cell *in situ* and can only measure target cell ratios from a large cell population (~10^5^ cells), without the capability of identifying single cells and directly counting exact number of target cells. Different from the device in reference [57] that detect target cell ratios via their average transit time change in a continuous flow, the device in reference [58] can capture target cells within the fluidic chamber via a layer of antibody functionalized magnetic beads. The number of total cells and non-target cells are counted separately using two micro Coulter counters; target cell number can thus be obtained by calculating the counts difference between the two counters. Although the device in reference [58] can count the target cells by deducting the counts of the non-target cells from the total counts of cells, it cannot detect single cells *in situ*; deposition of magnetic bead layer only in the fluidic chamber (capture chamber) is difficult to control. The above devices lack the capability of single cell analysis, a dramatic barrier for utilizing them [57,58] to advance the single cell research.

To overcome the above limitation, here we report a microfluidic magnet bead assay that can not only accurately identify the single cell *in situ*, but also measure the sizes of individual cells and accurately count the cells within a mixed population. With the compact size and simple operation steps, this method is capable of single cell detection in a continuous flow and enables cell reuse for downstream applications.

## Materials and methods

### Sensing principle

**Fig 1:**
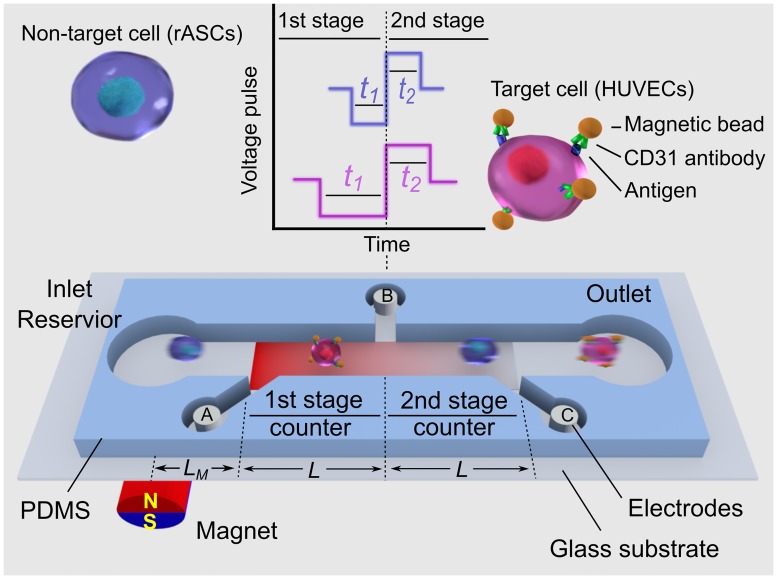
Schematic of the sensing principle of the magnetic bead assay for single cell detection. Not to scale.

To detect single cell in a continuous flow, we designed a microfluidic device that can identify and count single cells via their magnetic property difference. As shown in Fig 1, a straight microfluidic channel was divided into two Coulter counters with identical geometry by three electrodes A, B, and C. The voltage on the 2nd counter (*V*_*BC*_) is continuously measured and recorded. When a cell passes through the 1st counter, the resistance of the 1st counter increases, while the resistance of the 2nd counter remains the same. The current flow through the channel decreases because the total resistance increases, leading the voltage drop across the 2nd counter (*V*_*BC*_) to decrease. Similarly, when the cell passes through the 2nd counter, the voltage across the 2nd counter (*V*_*BC*_) increases. By monitoring the output voltage *V*_*BC*_, a cell passing through the two successive sensing channels generates one negative voltage pulse and one positive voltage pulses consecutively. The pulse width and the pulse height indicate the transit time and size of one cell respectively. In our previous works, cells can only be detected on a population basis, by measuring the cell counts difference [58] or the average transit time difference [57] between two separated micro Coulter counters. Here instead of measuring the bulk response from a large cell population, single cell response (individual paired voltage pulses) can be detected with two consecutive counters. Hence, the cell detection resolution can be significantly improved to single cell level.

To differentiate a target cell from non-target cells, an external magnet was mounted close to the 1st stage to create a higher magnetic energy gradient at the 1st stage than that at the 2nd stage counter (see Fig 1) to induce transit time change. Target cells were first conjugated with magnetic beads via specific binding between cell surface antigen and the corresponding antibody. A target cell conjugated with magnetic beads traveling through the sensing channel is subject to the magnetic force, which is proportional to the magnetic energy field gradient. Thus, the target cell is decelerated and has a longer transit time at the 1st counter than that at the 2nd counter (*t*_*1*_>*t*_*2*_). In comparison, a non-target cell travels through the two counters with the same transit time (*t*_*1*_ = *t*_*2*_), owing to no interaction with the magnetic field. Note that the transit time of a cell at the 1st stage counter could be increased by unintended flow fluctuation or non-specific interaction with microfluidic channel. Therefore, in addition to measuring the absolute transit time at the 1st counter, two consecutive counters are used to detect the transit time change of a single cell *in situ*. While cells passing through two consecutive counters are subject to the same flow fluctuation and nonspecific interaction, the differential measurement of (*t*_*1*_- *t*_*2*_) would minimize the flow fluctuation and non-specific interaction effects. Thus by measuring the two successive voltage pulses generated by each cell, we can identify each target cell *in situ* in terms of (*t*_*1*_- *t*_*2*_) without a need for the difficult surface modification of microchannels.

### Device fabrication and testing procedures

**Fig 2:**
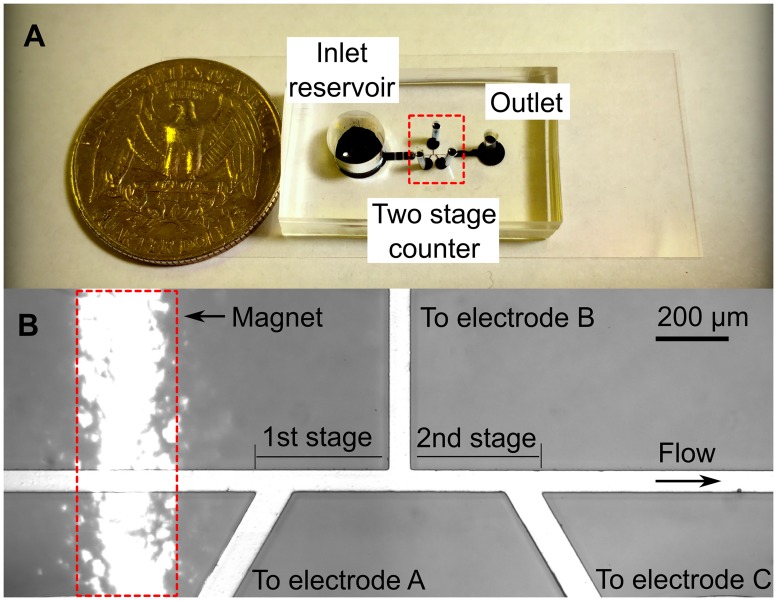
(A) Picture of the single cell detection device. (B) Microscopic image of the two stage counter structure.

We used standard soft lithography method to fabricate the compact microfluidics chip as shown in Fig 2(A). Briefly, we first created a SU-8 (2–25, MicroChem) master pattern consisting of a straight counter channel, three detecting arm channels where the electrodes are placed, one inlet reservoir, and one outlet. A PDMS (polydimethylsiloxane) slab was made by pouring the PDMS on top of the SU-8 master pattern, followed by degassing and curing the PDMS. Next, we created the interfaces for inlet reservoir, electrodes, and outlet by punching the PDMS slab with biopsy punches. Finally, the whole PDMS slab was bonded to a thin glass substrate (SuperSlips^™^ No.1, VWR, USA) after the air plasma treatment (200mTorr, 50W, 50 s). The nominal dimensions of the two successive Coulter counting channels are 60 μm wide (*W*), 35 μm deep (*D*) and 400 μm long (*L*) for both stages; the dimensions measured by the surface profilometer (Dektak 150, Veeco Instrument, USA) were 57.45 ± 3.16 μm wide, 36.16 ±0.03 μm deep, 396.23 ±1.77μm and 397.97 ± 1.37μm long for the 1st and 2nd counters. Three Ag/AgCl electrodes were inserted into the detecting arm channel to measure the voltage pulses from the two counters. An external cylindrical magnet (R063-063-DM, Amazing Magnet, USA) was mounted before the 1st stage counter, underneath the glass substrate, as shown in Fig 2(B). The inlet reservoir has a diameter of 6 mm and is 6 mm deep.

For each test, the cell suspension was loaded into the on-chip inlet reservoir and driven to pass through the device by a syringe pump at a flow rate of 900 μL/h. To reduce the sampling error, we mixed the concentrated cell suspension sample with cell culture medium in the inlet reservoir and maintained the cell concentration at 50 cells/μL for all the experiments. The two successive Coulter counters are modeled as two variable resistors (*R*_*1*_ and *R*_*2*_, see the supporting information). We applied a DC bias of 2V over the 1st and 2nd stage counter, such that 1V can be applied across each counter. The voltage output (*V*_*BC*_) was amplified by an external circuit and recorded by an NI-DAQ board (PCI-6133, National Instrument, USA) at a sampling rate of 600kHz. We used a custom Matlab^®^ program to analyze the recorded voltage output (*V*_*BC*_). The program first pairs the negative and positive pulses generated by the same single cell, and then measures pulse widths (*t*_*1*_ and *t*_*2*_) of the two successive pulses and calculate (*t*_*1*_*—t*_*2*_) to differentiate target cells from non-target cells. On the other hand, the pulse magnitude change (|δ*V*_*BC*_/*V*_*BC*_|) is proportional to cell volume (~*d*^*3*^), so that we can calculate cell diameter (*d*) by (Eq 1) [29,59]:

$$\left|\frac{\delta V_{BC}}{V_{BC}}\right|=\;\frac{d^{3}}{LD^{2}}\left[\frac{D^{2}}{2L^{2}}+\frac{1}{\sqrt{1+{\left(\frac{D}{L}\right)}^{2}}}\right]\;F\left(\frac{d^{3}}{D^{3}}\right)$$ (1)

where *V*_*BC*_ is the voltage output, and *L* is the channel length (400 μm). *D* is the characteristic channel diameter (which was estimated to be 51.43 μm according to *D* = (4 × *A*/π)^1/2^ for a square channel, where *A* is the cross-section area (2.08× 10^−9^ m^2^). *F* is the correction factor, which was taken to be 1 for calculation. [29]

### Ethics statement

This study was conducted in accordance with the regulations and approval of the Animal Care and Use Committee (IACUC) at the University of Akron (Protocol Number 14-11-14-ZRD) and in compliance with standards issued by the United States Department of Agriculture, Public Health Service and the American Association for Accreditation of Laboratory Animal Care. Adipose tissue was harvested from the animals euthanized with gas anesthesia, and all efforts were made to minimize suffering.

### Cell culturing and magnetic conjugation

Human umbilical vein endothelial cells (HUVECs; Lonza, Walkersville, MD, USA) were cultured in EGM-2 Bulletkit^™^ medium supplemented with 1% antibiotic-antimycotic (Gibco, Carlsbad, CA, USA). Rat adipose-derived stem cells (rASCs) were expanded in MesenPRO RS^™^ medium kit supplemented with glutamate (Gibco) and 1% antibiotic-antimycotic. Both cells were maintained in a humidified cell culture incubator at 37°C and 5% CO2 with media change every 2 days. All experiments were performed using HUVECs at passages 5–10 and rASCs at passages 2–5.

CD31 antibody functionalized Dynabeads^®^ (4.5μm diameter) were purchased from Life technologies. To minimize magnetic beads aggregation, the Dynabeads^®^ were first vortexed and then incubated on an orbital shaker (100 rpm) at room temperature for 15 minutes prior to magnetic beads conjugation. Next, the magnetic beads were conjugated to HUVECs following our optimized protocol as previously described. [57] Briefly, HUVECs were incubated with CD31 antibody functionalized Dynabeads^®^ at a ratio of 1:15 in 1 ml reaction volume at 4°C with gentle shaking for 30 minutes. The reaction products were then washed extensively using HUVECs culture medium to get rid of loosely bound magnetic beads. Then the Dynabeads^®^ conjugated HUVECs were subject to magnetic separation for positive cell selection to achieve the nearly 100% purity. The pure Dynabeads^®^ conjugated HUVECs were then mixed with rASCs to obtain mixed population with different cell mixing ratios, ranging 2% to 100%. Here the rASCs were used to represent other cell type in a heterogeneous cell population. The testing mixed cell populations were prepared at the concentration of 1000 cells/μL and diluted on the chip to 50 cells/μL for all the cell detection experiments. Phase contrast images of Dynabeads^®^ conjugated HUVECs were captured using the inverted AxioVision A1 microscope (Carl Zeiss, Oberkochen, Germany).

## Results and discussion

In this study, HUVECs were chosen as model testing cells because they have cell type specific antigen (CD31) expression and relatively uniform cell size. The specific antigen expression allows high affinity binding with antibody functionalized Dynabeads^®^. The relatively uniform cell size reduced any potential background noise during testing caused by the cell to cell size variation. In addition, HUVECs plays critical roles *in vitro* and *in vivo* angiogenesis and have very broad clinical applications. To better mimic a heterogeneous cell suspension, which is more realistic for future applications, we mixed HUVECs with rASCs. rASCs is another cell type commonly used for studying angiogenesis. They have similar cell size compared with HUVECs but do not express CD31.

### Device calibration

To ensure the microfluidic device can accurately measure the size and concentration of cells, we used standard polystyrene microparticles (74491 Fluka, Sigma-Aldrich) and cells to test on the device. The particle size measured by our device was 20.1 ± 0.5 μm, which agrees well with the manufacture specified size (20.0 ± 0.3 μm). We also tested the size distribution for the HUVECs and rASCs to be 18.7 ±3.5 μm and 17.5 ± 1.8 μm, respectively. Floating magnetic beads can be differentiated from cells by size. Note that in our previous work, [57] cells retained high viability after passing through a microfluidic channel. In this work, we used a wider channel, medium flow rates and suspended cells within mild cell culture medium, such that cells viability can be well maintained. For the concentration test, we used pure HUVECs (without magnetic beads conjugation) and diluted the cells to three different concentrations 13, 50 and 100 cells/μL. The measured concentrations were 13±2, 50±3 and 110±6 cells/ μL, indicating that the device can perform accurate detection within a wide concentration range. Note that the sensing channels are designed to be 60 μm wide, 35 μm deep and 400 μm long. Therefore, the two stage counter can only hold a tiny volume of sample of approximately 1.7 nL. Given the highest sample concentration at 100 cells/μL used in the testing, there is one cell in 10 nL fluid volume if cells are uniformly distributed, which is an order higher than the volume of the sensing channel. Thus the probability is very low for multiple cells passing through the sensing channel at the same time. Thereby every voltage pulse indicates the passage of each single cell. If the samples with higher cell concentration are tested, shorter sensing channels and larger magnetic field gradient should be used to reduce the volume of the sensing channels and generate sufficient transit time difference to achieve single cell detection resolution.

### Transit time analysis

**Fig 3:**
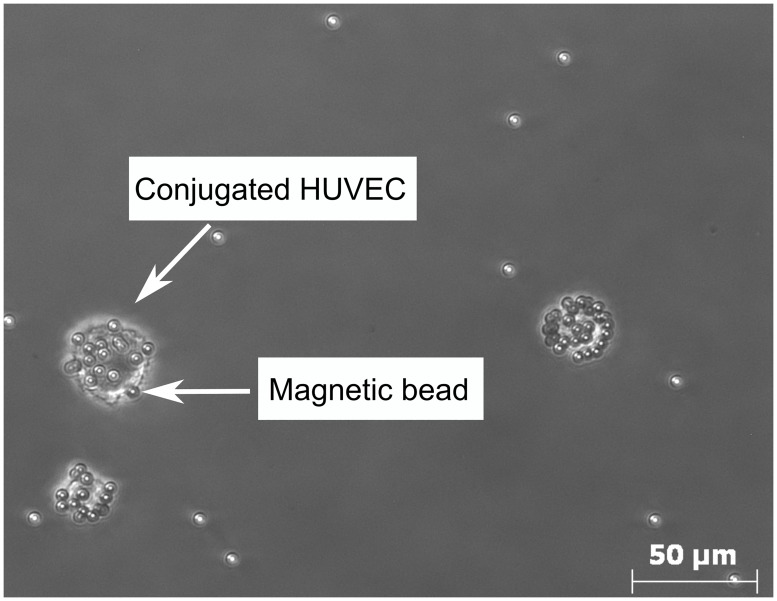
Microscopic image of the HUVECs conjugated with magnetic beads.

The HUVECs were conjugated with magnetic beads through specific antibody−antigen reaction. The conjugated HUVECs were observed under the microscope, as shown in Fig 3. The magnetic beads were functionalized with the CD31^+^ antibody, which can specifically bind to the CD31 antigen expressed on the cell membrane of HUVECs. We found the average number of beads conjugated with each HUVEC (*N*) was 17, which was close to the cell to bead ratio (1:15) we used for conjugation.

To analyze the transit time for a target HUVEC, we then used the average number of magnetic beads conjugated to each HUVEC (*N*), the magnetic energy gradient (∇***B***^*2*^), and magnetic properties of the beads for theoretical analysis (see the supporting information). From the analysis, we can estimate the average magnetic force applied to a single HUVEC. Based on the magnetic force and flow velocity, the HUVEC movements along vertical direction (*l*_*z*_) and transit time change (*t*_*1*_—*t*_*2*_) through the microchannel can be estimated under different flow velocities (*V*_*fx*_). Note that under a low flow velocity (*V*_*fx*_ < 0.1 m/s), although the transit time change of HUVEC can be increased, the HUVEC could be captured by the magnetic field if its vertical travel distance is larger than the channel depth (*l*_*z*_ > *D*). We calculated that when the flow velocity is from 0.1m/s and 0.2m/s, the conjugated HUVEC can be slowed down by the magnetic field along the flow direction, yet not being captured by the microchannel (please see supporting information for details).

From the experimental observations, we confirmed that under a flow velocity of 0.12m/s, no HUVECs were captured by the magnetic field. With this flow velocity (*V*_*fx*_ = 0.12m/s), the microchannel length (*L* = 400 μm) and the calculated magnetic force, we estimated the average transit time for a target HUVEC to be 6.55 ms and 4.34 ms at 1st and 2nd counters, with a transit time change of 2.21 ms; while for a rASC the transit time maintain the same around 3.33 ms.

### Cell detection from pure populations

**Fig 4:**
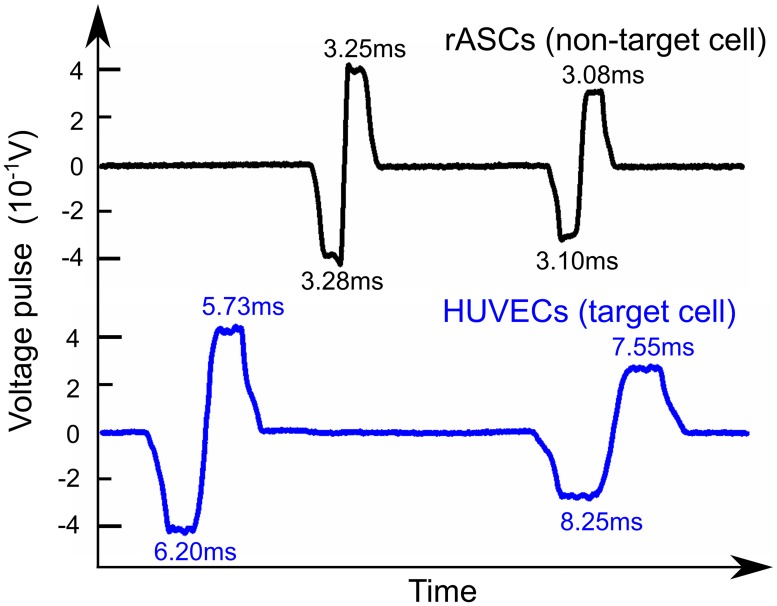
Typical pulses generated by target cells and non-target cells.

To experimentally validate the design concept of this method, pure magnetic bead conjugated HUVECs and pure rASCs groups were tested on the device separately and each cell’s transit times were measured. Under the same flow rate, voltage pulses generated from both cell groups were recorded and analyzed. The typical voltage pulses generated by HUVECs and rASCs are shown in Fig 4. It is obvious that a HUVEC has longer transit time at the 1st counter than that at the 2nd counter (*t*_*1*_—*t*_*2*_>0). In comparison, a rASC has nearly the same transit time as expected. Note that a HUVEC had longer transit time in both counter 1 and counter 2 because it was subject to magnetic forces in both counters.

**Fig 5:**
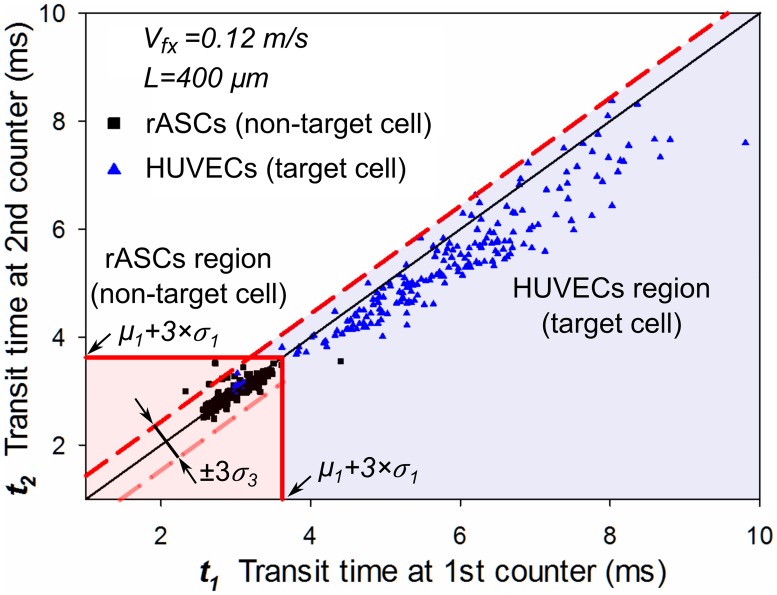
Cell transit time distribution of target and non-target cells.

To set up the identification criteria for target cells, we followed a similar method used in FACS to categorize different groups of cells into separate identification regions. Based on the measured transit time results from the pure rASCs and conjugated HUVECs groups, a scatter plot was drawn to illustrate the transit time of each cell at 1st counter (*t*_*1*_) and second counter (*t*_*2*_), as shown in Fig 5. 217 rASCs were counted and plotted as black points, while 203 HUVECs were counted and plotted as blue points. Fig 5 clearly shows two distinct cell populations (blue for target cells and black for non-target cells). Next identification regions for each cell type were set quantitatively.

We first set up a region for identifying the non-target cells (rASCs). Note that rASCs have significant shorter transit times than the HUVECs in both counters because there were no magnetic interactions between rASCs with the counters. For the transit time at the 1st counter, the rASC group has a significantly different distribution (*μ*_*1*_ = 3.00ms and *σ*_*1*_= *0*.*24ms*) than the HUVECs (*μ*_*2*_ = 5.72ms and *σ*_*2*_ = 1.42ms). Transit time of over 99.5% rASCs is less than the upper limit (*μ*_*1*_ + 3×*σ*_*1*_ = 3.72ms) from the distribution. Because transit times of rASCs through the two counters are nearly identical with the same standard deviation, we set a square region (*t*_*1*_< *μ*_*1*_ + 3×*σ*_*1*_, *t*_*2*_< *μ*_*1*_ + 3×*σ*_*1*_) as non-target cell identification region, seen as the light pink square. Within this region, over 99.5% of non-target cells (216/217) are included.

Note that transit times of some cells were subjected to flow fluctuation and off-axis effects,[60,61] and therefore had a small variation in transit time change (Δ*t* = *t*_*1*_—*t*_*2*_), even if there was no magnetic interaction. This was observed from the rASCs population in Fig 5. For the rASCs group, the mean (*μ*_*3*_) and standard deviation (*σ*_*3*_) of the transit time change (Δ*t*) were calculated to be 0.012ms and 0.136ms. Most non-target cells (213/216 = 98.6%) were distributed within the ±3×*σ*_*3*_ region. The flow fluctuation and off-axis effects should be taken into account to set up the target cell identification region.

Next, we set a target cell (HUVECs) identification region shown as the light blue region in Fig 5. Because HUVECs had a longer transit time at the 1st counter (Δ*t* = *t*_*1*_—*t*_*2*_>0), the majority of HUVECs were distributed on the right side of the reference line (central solid black line, *t*_*1*_ = *t*_*2*_). On the other hand, part of HUVECs were affected by flow fluctuation and off-axis effects, [60,61] and were distributed on the left side of the reference line (Δ*t* <0), which would lead to detection error by underestimating the total target cells number. Within the same flow conditions, these two effects would also affect non-labeled cells passing through the counters; the *Δt* change caused by these effects was regarded the same for both labeled and non-labeled cells. Therefore, to reduce the detection error, we can include those labeled cells affected by the flow fluctuation and off-axis effects, by using the criteria (*t*_*2*_—*t*_*1*_) < 3×*σ*_*3*_ obtained from the unlabeled cell test. Hence we set the target cell identification region to be the region outside the non-target cell region (*t*_*1*_ ≥ *μ*_*1*_ + 3×*σ*_*1*_, *t*_*2*_ ≥ *μ*_*1*_ + 3×*σ*_*1*_) and below the dashed red line (*t*_*2*_—*t*_*1*_) < 3×*σ*_*3*_, shown as the light blue region. Using these identification criteria, 187 cells out of 203 total counted cells (>92.1%) were identified as target cells. A small portion (7.9%, 16/203) of target cells were counted as non-target cells. The error is partly caused by the conjugated magnetic beads loss during the processes of dilution, transfer and flowing through the microfluidics channel. Nevertheless, by setting regions for both types of cells, we can differentiate target cells from non-target cells with high accuracy.

### Cell detection from mixed populations

Next, to demonstrate the validity of the magnetic bead assay for single cell detection under mimicked heterogeneous cell suspension environment, we tested five mixed cell samples with different target cell ratios (2%, 5%, 10%, 30% and 50%) with the device. For each target cell ratio test, we created two groups with the same HUVECs ratio: one treated group (conjugated HUVECs mixed with rASCs), and one untreated group (un-conjugated HUVECs mixed with rASCs). Here conjugated HUVECs were used to mimic the target cells, while rASCs and un-conjugated HUVECs were non-target cells. Each group was tested with the device separately.

**Fig 6:**
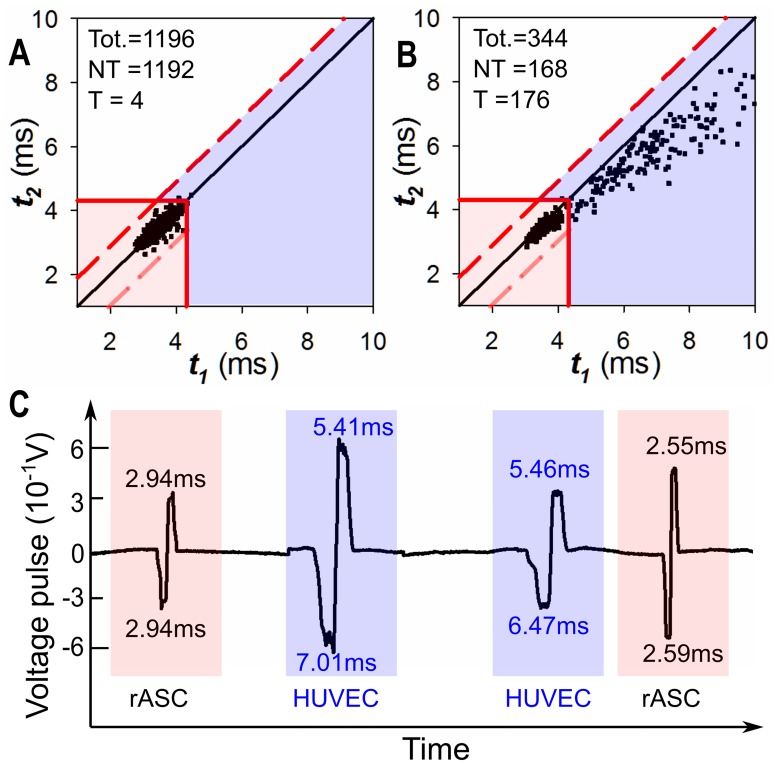
Testing results for mixed cell groups with 50% HUVECs ratio. (A) Cell transit time distribution of the untreated mixed group (un-conjugated HUVECs mixed with rASCs). From this distribution, the identification regions for non-target cell and target cell are set and shown as the pink and blue regions. (B) Cell transit time distribution of the treated mixed group (conjugated HUVECs mixed with rASCs). Using the identification regions, cells were categorized and counted (168 rASCs and 176 HUVECs). (C) Typical voltage pulses generated from the 50% treated mixed group.

We used the 50% mixed group as an example to illustrate the procedures to set up the identification region for identifying target cells (conjugated HUVECs). The procedures are similar to the procedures used in FACS to create the custom defined thresholds. Firstly, we ran the untreated mixed group (un-conjugated HUVECs mixed with rASCs) on the device as a control group. Because no magnetic beads were conjugated with HUVECs, the HUVECs within this mixed group were also regarded as non-target cells. Based on the measured transit time from this untreated mixed group, we calculated the maximum transit time of non-target cell (*μ*_*1*_ + 3×*σ*_*1*_ = 4.31ms), as well as the transit time change fluctuations (3×*σ*_*3*_ = 0.90ms). Afterwards, using the same method illustrated in Fig 5, we set up the identification regions for target cells (*t*_*1*_ ≥ 4.31ms, *t*_*2*_ ≥ 4.31ms, (*t*_*2*_—*t*_*1*_) <0.90ms) and for non-target cells (*t*_*1*_ < 4.31ms, *t*_*2*_ < 4.31ms), shown as blue and pink regions in Fig 6(A). From the untreated mixed group, 1192 out of 1196 cells (99.7%) fall into the non-target cell identification region.

Next, we tested the treated mixed group (conjugated HUVECs mixed with rASCs) on the device. Using the same identification region/criteria we set in Fig 6(A), target HUVECs and non-target rASCs can be identified. Fig 6(B) shows a scatter plot of every single cell from the 50% mixed groups, where 168 and 176 cells were counted as rASCs and HUVECs, respectively. The measured target cell ratio was then calculated to be 176/344 = 51.2%. The slight difference (1.2%) was possibly caused by non-uniformity and uneven mixing of the cell sample used in the experiment. Typical voltage pulses from the 50% treated mixed group in a continuous flow are also plotted in Fig 6(C). It is obvious that from the pulse widths (*t*_*1*_ and *t*_*2*_) and pulse width difference (*t*_*1*_—*t*_*2*_), each single conjugated HUVEC can be easily recognized *in situ* on a single cell basis. Note that the transit times of target cells can be affected by the flow fluctuation and off-axis effects, which are random and may differ from test to test. Therefore, slightly different target cell distribution can be found among groups (i.e. pure target cell test and mixed treated cell test) and tests. To minimize these effects in the future, cells can be pre-focused along the central stream line to reduce the off-axis effect; additionally, the flow fluctuation effect can be minimized by using more precise and stable flow control components.

**Fig 7:**
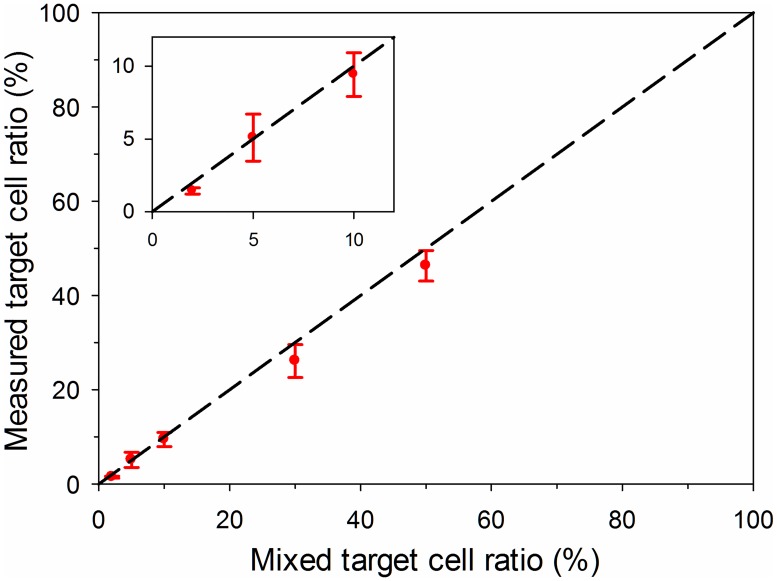
Testing results for the mixed cell groups with different target cell ratios (2%, 5%, 10%, 30% and 50%).

We used the same testing procedures to detect target HUVECs from other mixed cell groups and measured the corresponding HUVEC ratios. The measured target cell ratio is plotted in Fig 7. The dashed line in Fig 7 represents the equivalence of the measured target cell ratio and the pre-set mixed ratio. Fig 7 indicates the measured target cell ratios agree well with the pre-set mixed ratio. The slight difference was possibly caused by magnetic beads loss on a small amount of cells during the sampling and testing processes. For each mixed target cell ratio, standard deviations of the four replicates were plotted in Fig 7. The standard deviations were caused by non-uniformity from sample to sample. Note that in the test the mixed population was created by mixing the conjugated HUVECs with rASCs to demonstrate the design concept of this assay. For practical applications, target cell magnetic conjugation can be implemented more effectively within a cell mixture, by following the established protocols for high efficiency magnetic cell isolations.[37,62]

In summary, we have demonstrated the single cell detection method using HUVECs and rASCs. This method is expected to be used for general cell detection from mixed cell populations. This method can detect any types of cells by using two successive micro counters, as long as a specific antibody can be found. In comparison to other on-chip cell detection method based on transit time change, [35] because the transit time change (*t*_*1*_—*t*_*2*_) can be obtained *in situ* using two successive micro Coulter counters, single cells can be identified with much less signal overlapping. Comparing with the bulky and costly FACS system, this cell detection method utilizes a compact integrated device, can be easily maintained and recovered to avoid sample wasting and cross contamination.

Also, experiment results indicated the high specificity and sensitivity of our method. This device is based on magnetic beads labeling of the target cells via specific antibody and antigen binding. Unlike fluorescence based labeling, where dead cells could introduce uncontrollable background fluorescence due to unspecific binding or autofluorescence, magnetic beads labeling has been proved to be highly specific and sensitive.[63–65] The device is intended to be used for detecting and analyzing single cells from known cell types, particularly for identifying single cell level variance under certain conditions (e.g. development, disease, treatment). For known cell types, cell specific markers have been established.[66–68] In this study, we demonstrated the feasibility of using one specific cell marker for identifying one cell type. In our future work, multiple properties of cells will be measured by labeling multiple cell markers on one cell, using different magnetic beads with various properties. In addition, the voltage pulse widths, representing the transit time of each single cell passing through the micro counter, can be recorded and analyzed in real time with this device. The device has broader potential clinical applications, because the detection only requires a very small number of cells and can detect the variations at a single cell level. Thus, the device can be potentially integrated to pathological analysis for disease diagnosis such as cancer and degenerative diseases. In addition, it can be used as a quality control testing method for cell based therapeutics. For real clinical applications, when the samples contain multiple cell populations and/or the targeted cell marker has extremely low level of expression, the detection resolution of this method can be maintained by tuning the external magnetic field and magnetic beads properties. Specifically, stronger magnetic core material (such as CoFe_2_O_4_) with higher saturation magnetization, [69,70] larger particle size, [36,71] and permanent magnet with stronger residual magnetic (*M*_*s*_) [43,72] can be used to achieve sufficient magnetic force needed for the required resolution. In addition, we could also chemically modify the coating of the magnetic beads to introduce indirect labeling of the cells to further magnify the signals.[36,73] In addition, the subtle variation of transit time change between each single cell reflects the various density of specific antigen expressing of the given cell and also could be related to unique cell electrical and physical properties. In the future, we plan to correlate our measurements with unique cell properties by in-depth analysis of the output signals and detailed characterization of cell electrical and physical properties. The obtained relationship would enable novel and ultrasensitive markers for high efficient cell identification, which could be used to effectively identify cells at different development, differentiation and disease prognosis stages.

## Conclusions

In this article, we developed a new single cell detection method based on magnet bead assay; this device can not only accurately identify and count single cell *in situ*, but also measure the size of each single cell. Each cell flowing through the two successive micro Coulter counters generates two successive voltage pulses. A target cell, conjugated with magnetic beads, interacts with the magnetic field over the counter, creating a longer transit time at the 1st counter than that at the 2nd counter (*t*_*1*_ > *t*_*2*_). Hence every single target cell can be detected and counted *in situ*; a large number of cells can be accurately identified in a continuous flow. We validated the detection method using target HUVECs and non-target rASCs. We then demonstrated that the device can identify and accurately enumerate HUVECs from mimicked heterogeneous cell suspension environments with five different target cell ratios (2%, 5%, 10%, 30% and 50%). With the simple structure and easy operation, this single cell detection method can be potentially used for drug screening, stem cell population analysis, and other target cell identification applications.

## Supporting information

S1 Supporting Information This file includes the equivalent circuit for the single cell detection device. (PDF)

S2 Supporting Information This file includes the magnetic force and transit time calculation. (PDF)

S1 Fig The Equivalent circuit for the single cell detection device. *R*_*1*_ and *R*_*2*_ represent the resistance of the two successive Coulter counters. *R*_*3*_ = *R*_*4*_ = 500kΩ. The voltage output *V*_*BC*_ was amplified by the differential amplifier (AD620, Analog Device, USA) and output as *V*_*out*_.
(TIF)

S2 Fig (A) The configuration of the device. (B) The magnetic force applied to a HUVEC as a function of its location along the flow direction.
(TIF)

S3 Fig (A) The vertical movement of a HUVEC within the 1st and 2nd stage counter, under different flow velocities. (B) The transit time change of a HUVEC under different flow velocities.
(TIF)
