# Supplementary material for: In situ single cell detection via microfluidic magnetic bead assay
Source: PLoS One. 2017 Feb 21;12(2):e0172697. doi: 10.1371/journal.pone.0172697 (PMC5319813; doi:10.1371/journal.pone.0172697)
Supplement: S1 Supporting Information — (PDF) [file pone.0172697.s001.pdf]

Supporting Information

In situ single cell detection via microfluidic magnetic bead assay

Fan Liu, [†] Pawan KC, [§] Ge Zhang, [§] *and Jiang Zhe[†] *

[†] Department of Mechanical Engineering, University of Akron, Akron, Ohio 44325, United States

[§] Department of Biomedical Engineering, University of Akron, Akron, Ohio 44325, United States

* Correspondence should be addressed to jzhe@uakron.edu, ge10@uakron.edu

This document contains the equivalent circuit for the single cell detection device.

The equivalent circuit for the single cell detection device.

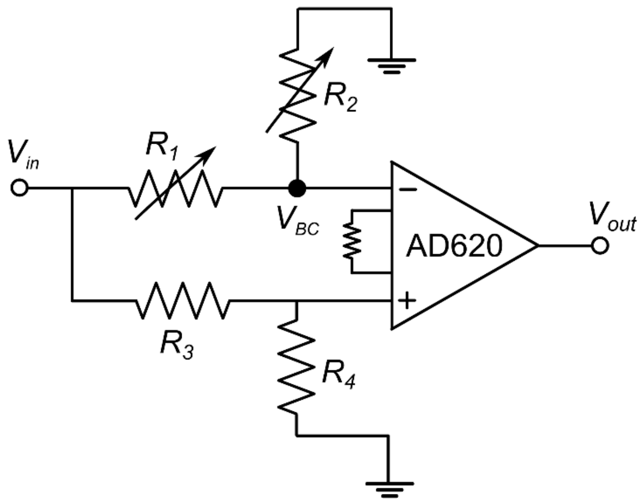

Fig S-1. The Equivalent circuit for the single cell detection device. R_1 and R_2 represent the resistance of the two successive Coulter counters. R_3=R_4=500kΩ. The voltage output V_{BC} was amplified by the differential amplifier (AD620, Analog Device, USA) and output as V_{out}.
