# Supplementary material for: In situ single cell detection via microfluidic magnetic bead assay
Source: PLoS One. 2017 Feb 21;12(2):e0172697. doi: 10.1371/journal.pone.0172697 (PMC5319813; doi:10.1371/journal.pone.0172697)
Supplement: S2 Supporting Information — (PDF) [file pone.0172697.s002.pdf]

Supporting Information

In situ single cell detection via microfluidic magnetic bead assay

Fan Liu, [†] Pawan KC, [§] Ge Zhang, [§] *and Jiang Zhe[†] *

[†] Department of Mechanical Engineering, University of Akron, Akron, Ohio 44325, United States

[§] Department of Biomedical Engineering, University of Akron, Akron, Ohio 44325, United States

* Correspondence should be addressed to jzhe@uakron.edu, ge10@uakron.edu

This document contains the magnetic force and transit time calculation.

Magnetic force and transit time calculation.

The magnetic force ($\boldsymbol{F}_m$) applied to each single conjugated HUVEC can be expressed as:

$$\boldsymbol{F_m} = N(\boldsymbol{m} \cdot \boldsymbol{\nabla})\boldsymbol{B} = N \frac{1}{2\mu_0} m_b \chi \boldsymbol{\nabla} \boldsymbol{B}^2 \quad (1)$$

where N is the average number of magnetic beads conjugated to each single HUVEC, which is estimated to be 17 from observation of conjugated HUVECs under a microscope. $\boldsymbol{m}$ is the magnetic dipole moment of the magnetic bead. Since the carrier fluid is non-magnetic cell culture medium with negligible magnetic susceptibility, the magnetic dipole moment can be written as $\boldsymbol{m} = m_b \chi \boldsymbol{H}$. Here, χ is the magnetic mass susceptibility, which is 16×10^{-5} m^3/kg. m_b is the weight of each magnetic bead, which can be estimated as 1.22×10^{-12} kg from the bead radius (4.5 μm) and density (1500 kg/m3). $\boldsymbol{B}$ is the magnetic flux density and can be expressed as $\boldsymbol{B} = \mu_0 \boldsymbol{H}$, where $\boldsymbol{H}$ is the magnetic field and μ_0 is the magnetic permeability constant equals to 1.26×10^{-6} N/A^2. Using the method described in our previous work,[1,2] the magnetic force along the flow direction can be estimated by equation below:

$$F_{mx} = N\mu_0 m_b \chi M_s^2 R_m^4 \frac{x'}{2[z'^2 + x'^2]^3} \quad (2)$$

where x' and z' represent the distances from the origin along flow direction and perpendicular to flow direction. Considering the magnetic radius (R_m) as 794 μm, glass substrate thickness as 160 μm and center position of the fluidic channel as 20 μm, we took z' as 974 μm. The cylindrical magnetic was magnetized to a level of M_s (0.99×10^6 A/m) through its radius direction. The configuration of the device is shown in Fig. S-2 (A). By using equation 2, we can estimate the magnetic force (F_{mx}) applied to each HUVEC according to its location (x') along the flow direction, as plotted in Fig. S-2 (B). By placing the magnet 500 μm before the 1st stage counter, the maximum magnetic force can be applied to a HUVEC at the 1st counter, so this configuration (L_M= 500 μm) was used for all of our experiments. The average magnetic forces applied to a HUVEC were then estimated to be to be 1.11×10^{-8} N and 5.37×10^{-9} N at the 1st and 2nd stage counter, respectively.

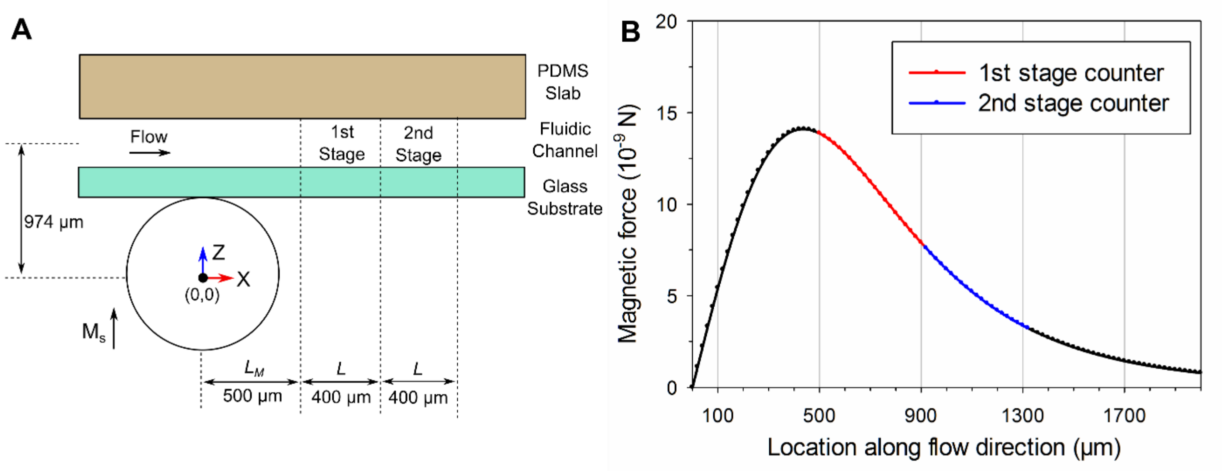

Fig S-2. (A) The configuration of the device. (B) The magnetic force applied to a HUVEC as a function of its location along the flow direction.

The Stock's drag force applied to each HUVEC is induced by the velocity difference between the cell and the fluid, and can be calculated by equation 3:

$$\boldsymbol{F_d} = 6\pi R_c \eta \Delta \boldsymbol{V} \quad (3)$$

where R_c is radius of the cell, an average value of 10.0 µm was used. η is the dynamic viscosity of the water, which equals to 1.00 × 10^{-3} (N·S/m^2). Along the flow direction, ΔV_x is the velocity difference (V_{fx}-V_{cx}) between the fluid (V_{fx}) and the HUVEC (V_{cx}). Along the vertical direction we assumed V_{fz} =0, because of the laminar flow, so that ΔV_z= 0-V_{cz}. Therefore, the trajectory of a HUVEC within the microfluidic channel can be solved using the first-order ordinary differential equations below:

$$\frac{dV_{cx}}{dt} = \frac{1}{m_c}\left[-F_{mx} + 6\pi R_c \eta \left(V_{fx} - V_{cx}\right)\right] \quad (4)$$

$$\frac{dV_{cz}}{dt} = \frac{1}{m_c}\left[F_{mz} + 6\pi R_c \eta \left(-V_{cz}\right)\right] \quad (5)$$

where m_c is the weight of conjugated HUVEC. Under different flow velocity (V_{fx}), HUVEC velocity can be solved, so that the transit time (t_1-t_2) and vertical movement (l_z) of a HUVEC were estimated and plotted in Fig. S-3 (A) and (B).

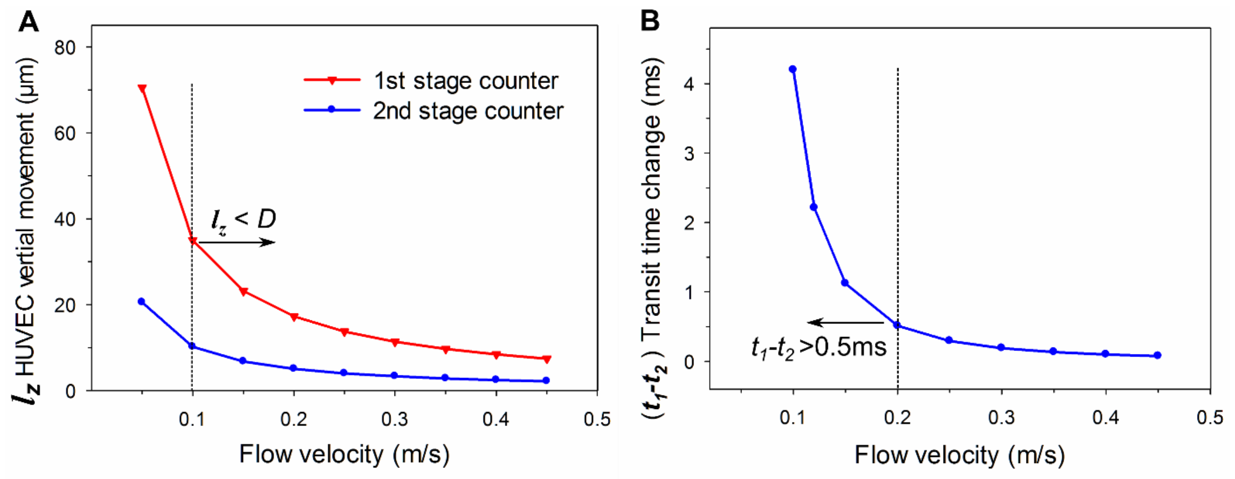

Fig S-3 (A) The vertical movement of a HUVEC within the 1st and 2nd stage counter, under different flow velocities. (B) The transit time change of a HUVEC under different flow velocities.

From Fig. S-3, we found that when the flow velocity is in the range of 0.1 m/s and 0.2m/s, a significant transit time change (t_1-t_2 >0.5ms) can be achieved and vertical movement of a HUVEC is limited (l_z < D =35 µm). And from the experimental observations, we further confirmed that under the flow velocity of 0.12m/s, no HUVECs were captured by the magnetic field. Therefore, based on this flow velocity (V_{fx} = 0.12m/s), the microchannel length (L= 400 µm) and the calculated magnetic forces, the HUVEC velocity (V_{cx}) at the 1st and 2nd stage can be calculated to be 6.10 ×10^4 µm/s and 9.21 ×10^4 µm/s; and the transit times of the HUVEC at the 1st and 2nd counter are estimated to be 6.55 and 4.34ms, with a significant transit time change of 2.21ms.

REFERENCES

1. Liu F, KC P, Zhang G, Zhe J. Microfluidic Magnetic Bead Assay for Cell Detection. Anal Chem. 2016;88: 711–717. doi:10.1021/acs.analchem.5b02716

2. Furlani EP, Ng KC. Analytical model of magnetic nanoparticle transport and capture in the microvasculature. Phys Rev E - Stat Nonlinear, Soft Matter Phys. 2006;73: 1–10. doi:10.1103/PhysRevE.73.061919
