# Supplementary figures and images for: In situ single cell detection via microfluidic magnetic bead assay
Source: PLoS One. 2017 Feb 21;12(2):e0172697. doi: 10.1371/journal.pone.0172697 (PMC5319813; doi:10.1371/journal.pone.0172697)

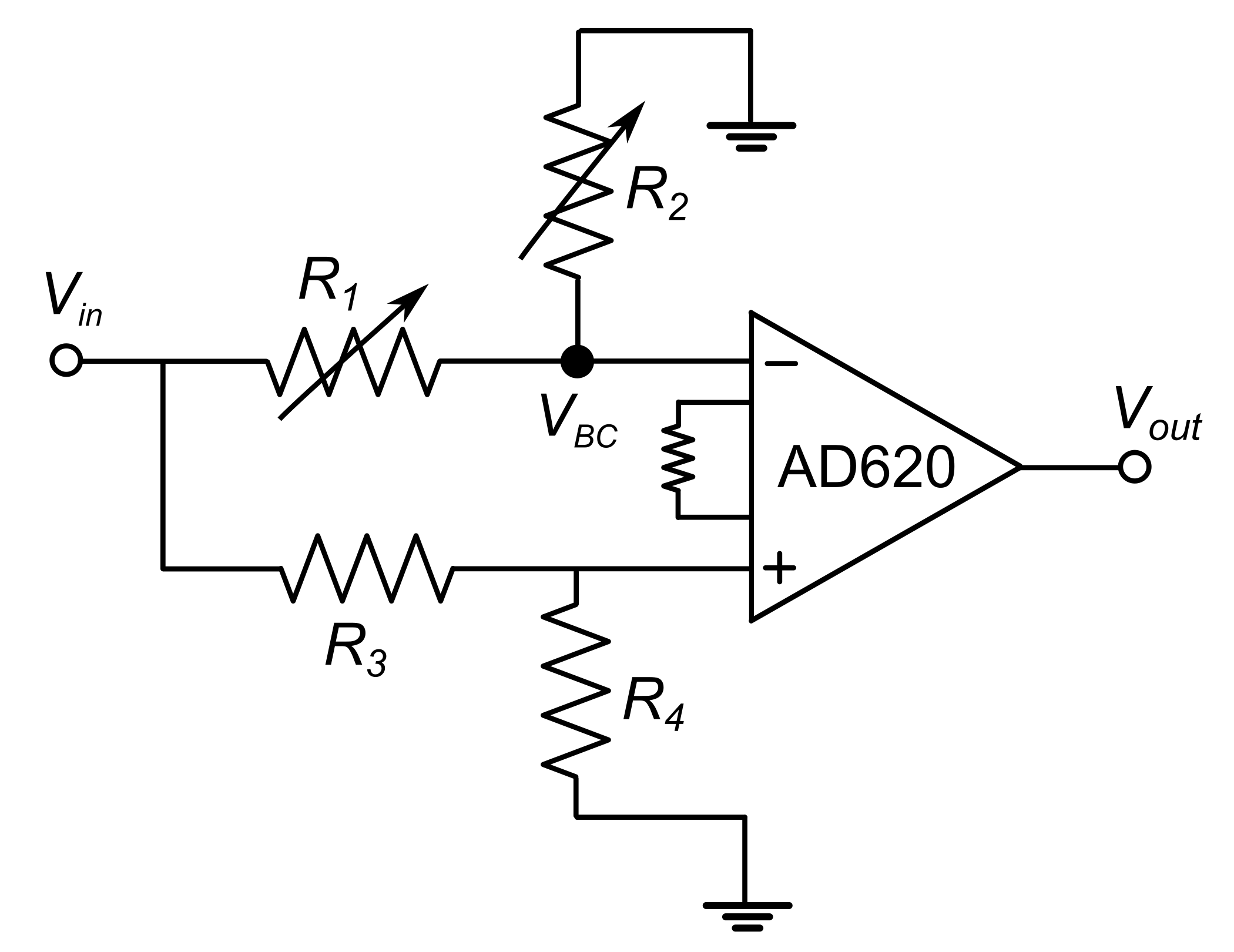

Supplement: S1 Fig — R1 and R2 represent the resistance of the two successive Coulter counters. R3 = R4 = 500kΩ. The voltage output VBC was amplified by the differential amplifier (AD620, Analog Device, USA) and output as Vout. (TIF) [file pone.0172697.s003.tif]

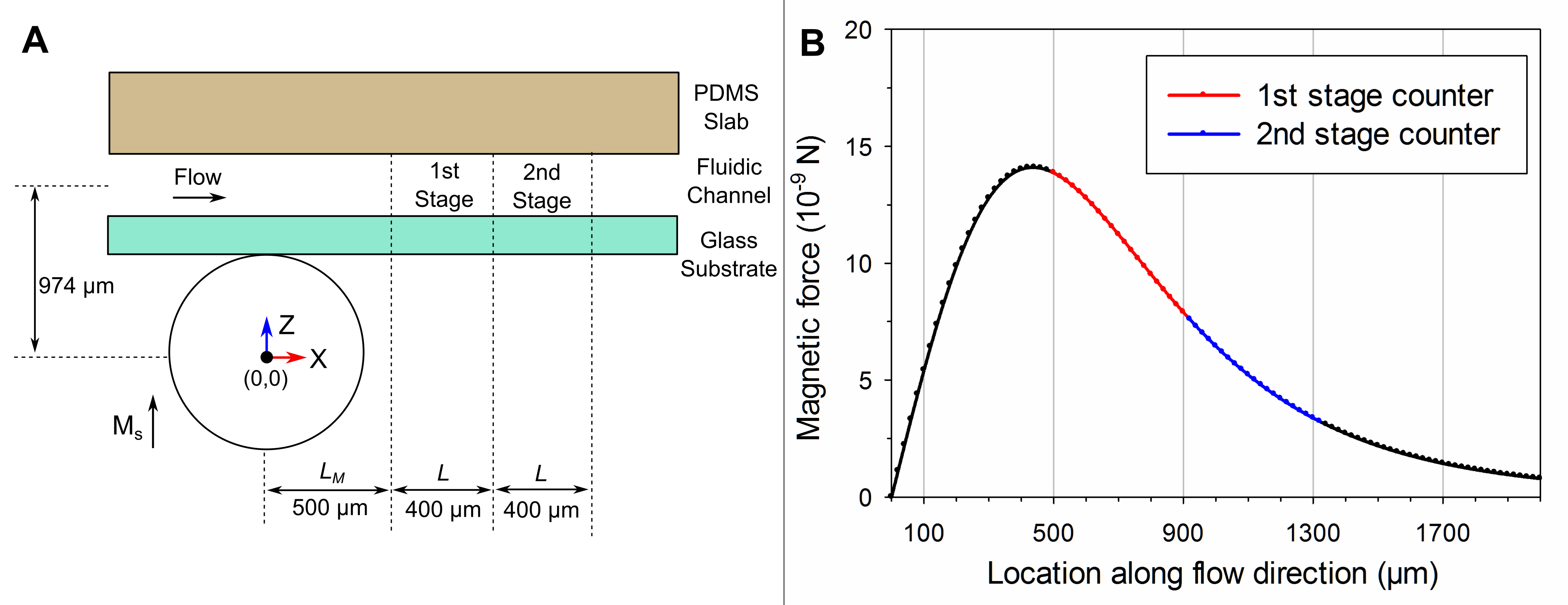

Supplement: S2 Fig — (A) The configuration of the device. (B) The magnetic force applied to a HUVEC as a function of its location along the flow direction. (TIF) [file pone.0172697.s004.tif]

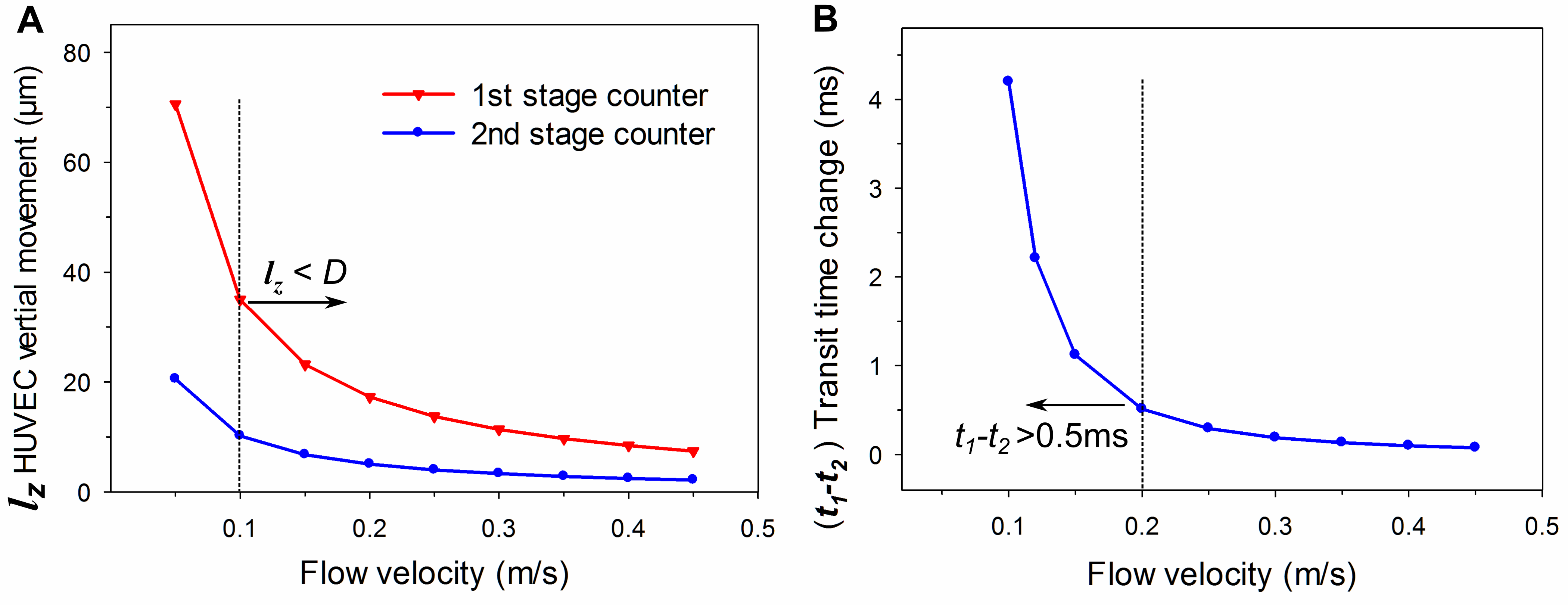

Supplement: S3 Fig — (A) The vertical movement of a HUVEC within the 1st and 2nd stage counter, under different flow velocities. (B) The transit time change of a HUVEC under different flow velocities. (TIF) [file pone.0172697.s005.tif]
